# Persistent reduced ecosystem respiration after insect disturbance in high elevation forests

**DOI:** 10.1111/ele.12097

**Published:** 2013-03-17

**Authors:** David J P Moore, Nicole A Trahan, Phil Wilkes, Tristan Quaife, Britton B Stephens, Kelly Elder, Ankur R Desai, Jose Negron, Russell K Monson

**Affiliations:** 1 School of Natural Resources and the Environment, University of Arizona, Biological Sciences EastTucson, AZ, 85721, USA; 2 Department of Ecology and Evolutionary BiologyRamaley N122, Campus Box 334, University of Colorado, Boulder, CO 80309-0334, USA; 3 School of Mathematical and Geospatial Sciences, RMITGPO Box 2476, Melbourne, Victoria, 3001, Australia; 4 Department of Meteorology and National Centre for Earth Observation, University of ReadingRG6 6BB, UK; 5 National Center for Atmospheric Research1850 Table Mesa Drive, Boulder, CO 80305, USA; 6 US Forest Service, Rocky Mountain Research Station240 W Prospect Road, Fort Collins, CO 80526, USA; 7 Department of Atmospheric & Oceanic Sciences, University of Wisconsin1225 W. Dayton Street, Madison, WI 53706, USA; 8 Laboratory for Tree Ring Research1215 E. Lowell Street, Box 210045, Tucson, AZ, 85721, USA

**Keywords:** Carbon balance, disturbance, ecosystem respiration, gross primary productivity, insect outbreak, lodgepole pine, mountain pine beetle, mountain West, subalpine forest

## Abstract

Amid a worldwide increase in tree mortality, mountain pine beetles (*Dendroctonus ponderosae* Hopkins) have led to the death of billions of trees from Mexico to Alaska since 2000. This is predicted to have important carbon, water and energy balance feedbacks on the Earth system. Counter to current projections, we show that on a decadal scale, tree mortality causes no increase in ecosystem respiration from scales of several square metres up to an 84 km^2^ valley. Rather, we found comparable declines in both gross primary productivity and respiration suggesting little change in net flux, with a transitory recovery of respiration 6–7 years after mortality associated with increased incorporation of leaf litter C into soil organic matter, followed by further decline in years 8–10. The mechanism of the impact of tree mortality caused by these biotic disturbances is consistent with reduced input rather than increased output of carbon.

## Introduction

Forest disturbance is a fundamental driver of terrestrial carbon cycle dynamics (Adams *et al*. 2010), but the long-term effects of different disturbance types are poorly characterised in land surface models (Running 2008). Initially, most disturbances shift an ecosystem to a carbon source, while recovery from disturbance is commonly associated with greater ecosystem carbon storage (Odum 1969; Magnani *et al*. 2007). The magnitude of the effects of disturbance and recovery on carbon sequestration can be as large or larger than the effects of climate; two-thirds of the total annual US carbon sink can be attributed to intensive forest management and agricultural abandonment (Schimel *et al*. 2000). High altitude forests in recovery from historical logging and managed for some degree of fire suppression are responsible for the majority of carbon uptake and storage in the Western United States (Pacala *et al*. 2001; Schimel & Braswell 2005). Since the early 1990s, bark-beetle infestations have been responsible for catastrophic levels of tree mortality across millions of hectares of forest in Western North America (Kurz *et al*. 2008; Raffa *et al*. 2008). The majority of the damage originates from one species, the native Mountain Pine Beetle *Dendroctonus ponderosae* (Man 2010). This outbreak occurs within the context of a multi-decadal general increase in tree mortality across the Western United States and globally (van Mantgem *et al*. 2009; Allen *et al*. 2010); where climate and hydrological change coupled with changing and increasingly susceptible forest conditions are likely drivers (Breshears *et al*. 2009). Phloem-feeding insect outbreaks reduce ecosystem productivity through tree mortality (Flower *et al*. 2012; Hicke *et al*. 2012) and the predicted impact of this most recent mountain pine beetle outbreak is the transition of western North American forests from a sink to a substantial source of carbon to the atmosphere (Kurz *et al*. 2008). Our findings do not support this prediction.

Modelling how carbon cycling will respond to these widespread infestations remains challenging because ecosystem-scale CO_2_ fluxes are notoriously hard to monitor in mountain forests and empirical results are only available over short temporal spans and small spatial scales (Morehouse *et al*. 2008; Amiro *et al*. 2010; Brown *et al*. 2010). Eddy covariance is limited in scale and subject to errors associated with complex flows (Yi *et al*. 2008; Sun *et al*. 2010); satellite observations have only been validated against carbon uptake terms (Heinsch *et al*. 2006; Sims *et al*. 2008); and chamber studies are labour intensive and difficult to scale. We present a rare set of complimentary observations to characterise the response of both photosynthesis and respiration in a forest unaffected by the beetle outbreak (Niwot Ridge; NWT) and an 84 km^2^ forested valley that has been markedly impacted by the outbreak (Fraser Experimental Forest; FEF). These data include locally calibrated satellite estimates of gross primary productivity (GPP), atmospheric CO_2_ monitoring that reflects nightly valley-scale respiration, and spatially distributed soil chamber flux and soil organic carbon measurements representing a decade long chronosequence since natural and manipulative disturbance. Collectively, these measurements and analysis products showed that forest respiration sharply decreased in the first 3 years after beetle disturbance, rebounded by year 6, and then declined again from 7 to 9 years post-disturbance.

## Materials and Methods

### Site descriptions

**Figure 1:**
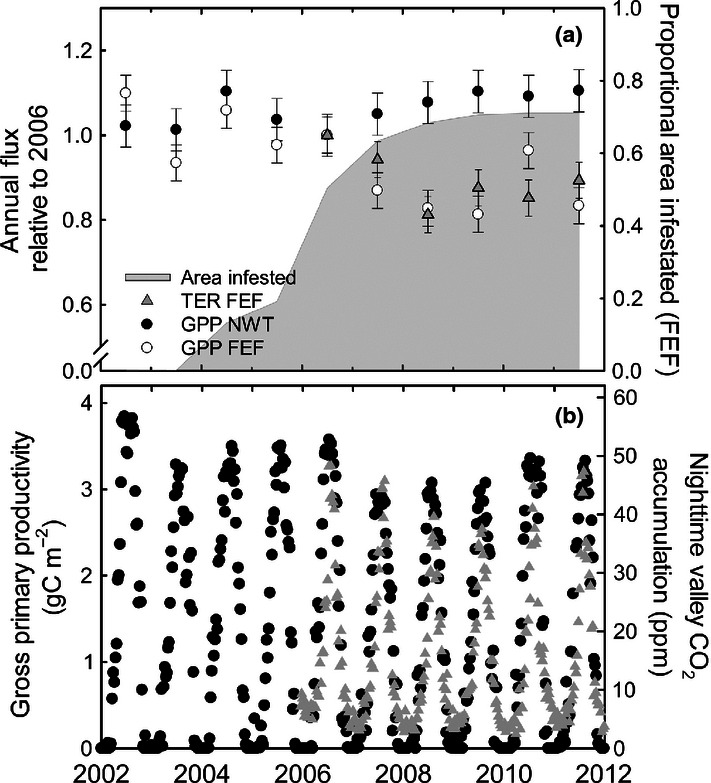
Annual estimates of valley-scale gross primary productivity (GPP) and total ecosystem respiration (TER). (A) The relative annual GPP at the un-infested Niwot Ridge (filled circles) and the infested Fraser Experimental Forest (FEF; open circles) and the coordinated decline in annual TER at FEF (grey triangles) starting in 2006. The shaded area represents the cumulative proportion of the St. Louis Valley classified as infested with mountain pine beetle (MPB). (B) Seasonal patterns in GPP and night-time valley CO_2_ accumulations (both 8-day averages) at FEF used to calculate the relative annual fluxes. Error bars represent standard errors of the least squared mean.

The FEF is a 93 km^2^ research forest maintained by the USDA located within the 1789 km^2^ Sulphur Ranger District, Arapaho National Forest, Colorado. Our chronosequence sites are located at 39°54′27″ N 105°51′10″ W at 2913 m in the lodgepole pine (*Pinus contorta* Dougl. ex Loud.) dominated subalpine forest which composes about half of the tree cover in the district. Engelmann spruce (*Picea engelmannii* [Parry]), and subalpine fir (*Abies lasiocarpa* [Hook.] Nutt. var. lasiocarpa) make up an additional quarter of the tree cover, predominantly at higher elevations, on north slopes, and along streams (Popovich *et al*. 1993). The remainder of the forest is made up of mixed tree species. The forest occupies the St. Louis Creek valley and several tributaries, with 850 m of vertical relief between ridge tops and valley bottom. Most of the forest became established after a stand-replacing fire in 1685, and low elevation portions, including our chronosequence sites were logged in the early 1900s. Annual precipitation averages 737 mm with about two-thirds falling as snow from October to May and the average annual temperature at 2745 m of 0.5 °C. Soils are typically derived from gneiss and schist with angular gravel, stone, very little silt and clay, and a thin organic horizon of ∼3–12 cm. Due to their permeability, these soils have a high capacity to store considerable water during snowmelt. More information can be found at http://www.fs.fed.us/rm/fraser/. Since 2002, approximately 70% of the St Louis Creek Valley study area has been infested with mountain pine beetle (MPB; Fig. 1). Infested trees were distributed throughout lodgepole pine subalpine forest (personal observation) and we established research plots within this area.

The Niwot Ridge (NWT) AmeriFlux site is located at 3050 m in a subalpine forest just below the Continental Divide near Nederland, Colorado (40° 1′ 58″ N; 105° 32′ 47″ W). The site encompasses an elevational ecotone between lodgepole pine and spruce-fir forest types, where 46% of tree cover consists of subalpine fir, 28% Engelmann spruce and 26% lodgepole pine. The area in the footprint of the Ameriflux site has experienced less than 6% infestation by beetles. The area surrounding the AmeriFlux tower was also logged in the early 1900s and shares similar stand size and structure with the FEF site. Annual precipitation for the site averages 800 mm (approximately 65% falling as snow) and the mean annual temperature is 1.5 °C. The soils are sandy inceptisols derived from granitic moraine with a thin organic horizon ranging from ∼ 3 to 9 cm. Eddy covariance fluxes, storage fluxes and estimation of NEE have been recorded for twelve consecutive years at the primary AmeriFlux tower (data can be accessed at http://urquell.colorado.edu/data_ameriflux/).

### Establishment of mortality chronosequences at FEF and NWT

We selected clustered stands of *P. contorta* killed by mountain pine beetle (MPB) at the site located in the FEF. One hillside slope with an age gradient of beetle infestation was selected for plot establishment to minimise chronosequence spatial, stand age and parent substrate variability. The plots spanned an approximate 25-hectare area, with plot elevations ranging from 2884 m to 2909 m, slopes from 3 to 13^o^ and the aspects from 15 to 331^o^.Stands were aged to within approximately 1–2 years since time of beetle infestation by the degradation status of the crown (Klutsch *et al*. 2009). Stands of three to five lodgepoles in similar states of crown decay were binned into three age classes, and plots of approximately 9–15 m^2^ were established around the dead pines, excluding live trees at a distance of at least 2 m. We set up a total of eighteen plots, three per infestation age class and six control plots of equivalent size, each with 3–5 live uninfested lodgepole pines of similar medium size diameter at breast height (dbh), about 10–20 cm. The control plots were geographically distributed throughout the site.

At the Niwot Ridge AmeriFlux site, previous researchers investigating tree rhizodeposition and soil respiration (Scott-Denton *et al*. 2006; Weintraub *et al*. 2007) created plots of *P. contorta* that were girdled in 2002, 2003 and 2004 or left alive as controls. We also girdled plots of *P. contorta* for a prior experiment in 2008, 2009 and 2010. Plot trees were girdled using a saw to make two parallel incisions approximately 15 cm apart around the circumference of the tree at breast height, and phloem and cambial layers were scraped away. Any apparent re-growth of cambial tissue was removed two weeks later. Girdled plots from 2008 and prior were trenched around the perimeter from 15 to 30 cm in depth to help minimise rhizospheric inputs from outside the plot. This study allowed the creation of an eight-year girdling disturbance chronosequence. Three forest plots about 20 m^2^ containing between three and twelve medium dbh class lodgepole were established or re-established for each year plots were girdled, along with six live tree plots for controls, for a total of twenty-four plots. Plots spanned an elevation of 3016–3028 m, a slope of 4.7–6.7^o^ and an aspect of 78–94^o^ over an approximate 5.5 ha area.

### Soil respiration measurements and extractable dissolved organic carbon analyses

After the sites became snow-free in mid-June of 2010, we measured soil respiration fluxes, and collected soil from the organic horizon for measuring extractable dissolved organic carbon (DOC). We present DOC data here as the water-soluble C component of soils originates in part from soluble carbohydrates and microbial biomass (Ghani *et al*. 2003) and therefore DOC is a better proxy for mineralisable C than soil total C which includes more recalcitrant physically and chemically protected C. We sampled at both sites at approximate two-week intervals from June 21^st^ to September 29^th^. On each occasion soil respiration was measured in four permanently installed polyvinyl chloride collars per plot using LI-COR LI-6400s equipped with 6400–09 soil CO_2_ flux chambers (LI-COR Incorporated, Lincoln, NE, USA). Soil samples were collected from random locations in the organic horizon of each plot and stored in a cooler on ice for return to the lab. Within twelve hours, we sieved and homogenised soils, removing coarse plant litter, debris, and larger (> 1 mm) roots. A solution was immediately extracted from the soils with 0.5m K_2_SO_4_ for nutrients and frozen at −20 °C until analysis. We quantified DOC using the non-purgable-organic-C protocol on a Shimadzu total organic carbon analyzer (TOC 5000, Shimadzu Scientific Instruments, Inc., Columbia, MD, USA).

Soil efflux, extractable DOC pools, litter and organic horizon depth were evaluated for the significance of main effect of time since disturbance by repeated measures mixed model anova (SAS proc mixed; The SAS Institute, Cary, NC, USA), with random variation at the plot level. Where appropriate Tukey-Kramer post hoc comparisons of time since disturbance classes were made (α = 0.05). Unadjusted seasonal averages and uncertainties for both soil efflux and total organic carbon are shown in Table S3.

### Net ecosystem exchange measurements and flux based GPP

Net ecosystem exchange (NEE) was measured using the Eddy Covariance technique as described previously (Monson *et al*. 2002). Briefly, the 30 min, time-averaged, lag-corrected, covariance between the CO_2_ concentration and turbulent fluctuations for vertical wind speed measured at 10 m above the canopy were added to the CO_2_ stored below the canopy with common corrections applied to estimate the NEE. The concentration of CO_2_ was measured using an infrared gas analyzer (model LI-6262, LI-COR Inc., Lincoln, NE, USA), and wind speed was measured using a sonic anemometer (model CSAT-3, Campbell Scientific Inc., Logan, UT, USA). Air temperature, relative humidity and atmospheric saturation vapour pressure deficit (VPD) were measured at two heights (2 m and 21.5 m) on the main tower (http://urquell.colorado.edu/data_ameriflux/) and a heated tipping bucket rain/snow gauge with a datalogger (Campbell Scientific, Logan Utah, model 23X) was used to measure precipitation.

To estimate GPP using fluxes, the exponential temperature response to total night-time ecosystem respiration was first estimated from night-time values of NEE during non-stable conditions (where u* was greater than 0.2 ms^−1^). Using this static temperature relationship, total ecosystem respiration was removed from daytime NEE to estimate GPP. Although there are many different approaches to calculate GPP from flux observations, most provide statistically equivalent estimates and we chose this method because there are few and relatively simple assumptions associated with it (Desai *et al*. 2008).

### Satellite based estimate of GPP

We combined satellite reflectance and eddy covariance measurements to estimate GPP using the Temperature and Greenness (TG) model (Sims *et al*. 2008). This model uses EVI (the Enhanced Vegetation Index, a measure of the amount of green vegetation) and LST (Land Surface Temperature) from NASA's MODIS sensors to calculate estimates of GPP removing the need for re-analysis or downscaled meteorological data (Sims *et al*. 2008) that is typically required in other satellite derived estimates of GPP. All MODIS data used in this research were accessed directly from the NASA ftp site: http://ftp://e4ftl01u.ecs.nasa.gov/. MODIS EVI (MOD13A2) and Land Surface Temperature (LST, MOD11A2) data were filtered using the MODIS QA bits and only the highest quality retrievals were retained. The removed data were then replaced using the mean of a 41-day temporal window surrounding the missing observation (Fig. S3). To examine the long-term (interannual) effects of the MBP on EVI and to remove unrealistic high frequency fluctuations caused by residual atmospheric and bi-directional reflectance effects, we used the annual mean EVI for each site as an input into the TG model (Fig. S2). To avoid biases caused by residual cloud, anomalously low values (EVI < 0.12) were removed from the calculation of the annual mean EVI.

We reformulated the TG model, preserving the response of the original function but with parameters with an intuitive meaning which can be estimated for a given location. The original formulation is GPP = EVI' × LST' × m, where m is an empirical scale factor and EVI' is adjusted to ensure zero GPP at 0.1 EVI units (Sims *et al*. 2008) given by EVI' = EVI-0.1, where EVI is the value of the enhanced vegetation index from the MODIS product (MOD13A2). The control of temperature on photosynthesis is given by, LST' = min[(LST/30), (2.5—0.05.LST)], where LST is the land surface temperature as observed by MODIS. Our alternative formulation restates LST' as, LST' = min[(LST-minLST)/(optLST-minLST), (maxLST-LST)/(maxLST-optLST)], where minLST is the lowest MODIS observed temperature at which photosynthesis takes place, maxLST the highest, and optLST the temperature at which photosynthesis is not limited by temperature.

To capture the response of the forest to very low temperatures typical of mountain forests we recalibrated the model specifically against data from the Niwot Ridge flux. Using the original model parameters as prior conditions (0 °C, 30 °C and 50 °C; specifying a normal distribution with a standard deviation of 5 °C in each case), we implemented a Metropolis-Hastings (MH) algorithm with a chain length of 2 00 000. To initialise the algorithm, we approximated an initial uncertainty for GPP values from eddy covariance as 0.1 gC/m^2^/day and generated a ‘burn-in’ set of 10 000 parameter proposals that are discarded. The converged chain was thinned by a factor of 20 to produce summary statistics. The parameter values were selected by finding the closest chain member to the mean of the resulting multivariate distribution. This resulted in a lower optimal (22 °C) and minimum temperature (−0.9 °C) of operation for photosynthesis than the original formulation. The annual totals for GPP estimated by the TG model (GPP_TG_) are normalised to 2006 (Fig. 1). The initial guess for GPP uncertainty overestimates uncertainty during the winter and underestimates uncertainty in the summer and is only used in the model calibration. The new TG model parameters were validated against the NWT GPP data for 2009 which were excluded from the calibration (Fig. S5; *P* < 0.01, *R*^2^ = 0.81). This leads to a slightly larger uncertainty (∼ 19%) than reported for a range of GPP partition methods (10–16%; Desai *et al*. 2008).

### Estimation of relative changes in valley respiration from night-time accumulation of CO_2_ at FEF

To estimate the dynamics of ecosystem respiration through time for the St. Louis Creek valley we measured the night-time accumulation of atmospheric CO_2_ concentrations at the valley base (Headquarters meteorological tower; 39º 54′ 25″ N 105º 52′ 58″ W, 2745 m). We use the night-time accumulation of CO_2_ as a measure of ecosystem respiration over the major portion of the valley upwind of the Headquarters site. To avoid unsubstantiated assumptions required to calculate flux magnitudes without high-resolution atmospheric modelling and more extensive wind observations, we do not attempt to estimate absolute flux numbers. Rather, we present a robust quantification of the temporal dynamics of the relative magnitude of respiration by directly measuring the background-corrected night-time build-up of CO_2_ in the valley. Additional analyses illustrating these steps are included in Appendix S1. Briefly, measurements were made at 2.5 min intervals at 3 heights using an NCAR AIRCOA system (National Center for Atmospheric Research, Automated Inexpensive Robust CO_2_ Analyzer (Stephens *et al*. 2011). Four calibration gases tied to the WMO CO_2_ scale are analysed every 4 h, while a single calibration gas is analysed every 30 min. At night-time, during moderate regional wind forcing, radiative cooling consistently leads to a stable drainage flow pattern (Fig. S5), characterised by down-valley and down-slope flows, with vertical mixing within the valley supported by axial rotor circulations (Pypker *et al*. 2007a,b).

We have identical AIRCOA instruments at the Niwot Ridge site (NWT, 3523 m, 30 km to the NE) and at Storm Peak Laboratory (SPL, 3210 m, 95 km to the NW). These sites typically experience descending clean-air conditions at night and we use their average as a measure of the background concentration entering the St. Louis Creek valley. Using both NWR and SPL allows for coverage during non-overlapping gaps in either data set, and provides a more regionally representative estimate of background concentrations.

To construct the interannual CO_2_-buildup time-series shown in Fig. 1, we used a similar technique to Pypker *et al*. 2007a,b). We calculate nightly (0000 to 0400 Local Standard Time) average CO_2_ concentrations from the highest inlets for each of FEF, NWT, and SPL, filtering at NWT and SPL for stable CO_2_ conditions with hourly standard deviations less than 1 ppm (Fig. S7). We also calculate nightly averages over the same 4-h period for data from the Headquarters and Fool Creek (3100 m, 3 km to SE) meteorological towers.

We subtract the nightly mean CO_2_ concentration of NWT and SPL from FEF. Finally, we select for stable drainage conditions at FEF by filtering for nights when: 1) wind direction at Headquarters is between 160 and 270 degrees (91% of nights), 2) wind direction at Fool Creek is between 170 and 230 degrees (77% of nights), 3) wind speed at Headquarters is less than 0.6 m/s (89% of nights), 4) wind speed at Fool Creek is less than 2.2 m/s (99% of nights) and 5) the relative variability in hourly mean CO_2_ build-up is less than 200% (88% of nights). Collectively, these filters remove 34% of the nights from the time-series.

We use the resulting stable drainage flow CO_2_ build-up values as a measure of soil respiration over the major portion of the valley upwind of the Headquarters site. To avoid unsubstantiated assumptions required to calculate flux magnitudes without high-resolution atmospheric modelling and more extensive wind observations, we do not attempt to estimate absolute flux numbers. Rather, we present a robust quantification of the temporal dynamics of the relative magnitude of ecosystem respiration by directly measuring the background-corrected night-time build-up of CO_2_ in the valley.

## Results

We found a sharp decline in GPP at the infested FEF from 2006 onwards, corresponding to the peak of beetle infestation, but no corresponding decrease at the non-infested NWT (Fig. 1a), with the time series for the two sites diverging by 13–30%. Ecosystem respiration at FEF appeared to decline after 2006 (Fig. 1a and b). Relative to 2006, GPP declined on average by 13.8% and apparent respiration by 12.4%, leading to a modest reduction in carbon sink capacity. Furthermore, while respiration declined approximately 19% by 2008, respiration then increased slightly in 2009 and remained approximately 12% lower than 2006 through 2011. GPP showed a steady decline through 2009 and oscillated in 2010 and 2011(Fig. 1a).

**Figure 2:**
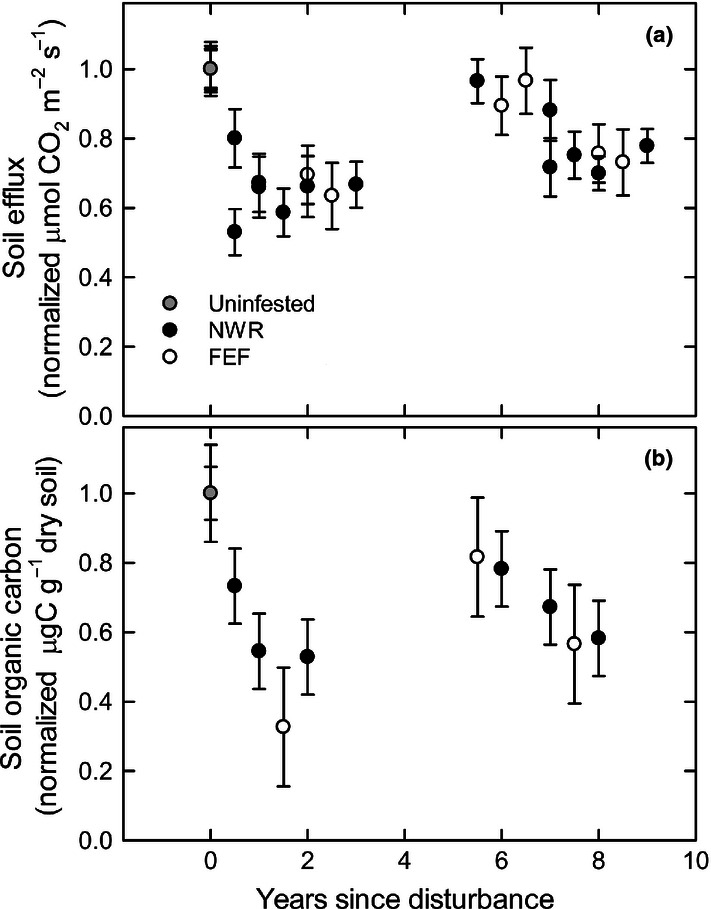
Tree mortality chronosequence plot soil respiration and carbon pools. Normalised soil CO_2_ efflux (μmol CO_2_ m^−2^ s^−1^; A) and extractable soil dissolved organic carbon (μg C g^−1^ dry soil; B) as a function of time since disturbance in FEF (open) and the NWT (closed symbols). At FEF the disturbance was caused by tree mortality induced by mountain pine beetle while at NWT trees were killed by manual girdling. Values are normalised to undisturbed plots (grey filled), and time since disturbance is estimated for FEF at ± 1-2 years. Errors represent standard error of the least squares mean.

Mean growing season soil respiration estimates from the mortality chronosequence plots changed significantly with time since disturbance for both the FEF beetle kill (*F* = 9.00, *P* < 0.05) and NWT girdling (*F* = 2.89, *P* < 0.05) chronosequences (Fig. 2a). There was a strong decline in measured soil respiration in plots containing killed trees over the first three years after disturbance as compared to plots with undisturbed lodgepole pines (33% NWT; 40% FEF). This decline was followed by a near complete recovery of respiration in plots ∼ 6 years post-disturbance (89% NWT; 96% FEF) lasting for 1–2 years, and a subsequent decline in respiration rates in the oldest mortality plots lasting out to 9 years after disturbance (Fig. 2a). The partial recovery of respiration did not exceed respiration rates in plots with live lodgepole pines, and corresponded with a pulse of fallen needle litter from dead trees. At FEF, litter and soil organic horizon depths were greatest in plots with beetle-killed trees 5–6 years post-disturbance (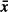 = 7.56 ± 0.63 cm), significantly higher (*F* = 4.25, *P* < 0.05) than in plots with live trees (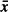 = 4.98 ± 0.51 cm) or recently killed trees (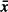 = 4.71 ± 0.65 cm) that still retained their needles. Soil extractable DOC first declined and then increased after 5–6 years at both sites (FEF, *F* = 3.47, *P* < 0.05; NWT, *F* = 3.53, *P* < 0.05; Fig. 2b).

## Discussion

Insect infestation caused a decline in respiration measured at scales from a few metres to an entire valley (84 km^2^). Although there was a partial recovery of respiration at the plot level, this was short-lived and likely associated with a temporary pulse in needle and possibly root litter. In contrast to the presumed mechanism governing carbon cycling after insect outbreaks, the patterns of GPP and respiration observed here, over nearly a decade post-disturbance, indicate that because of a decline in new substrate for respiration (GPP), the immediate impacts of the current beetle outbreak in North America on carbon release are likely to be lower in magnitude than previous estimates.

In most ecosystems, nearly half of the CO_2_ released in soil respiration derives from living plant roots, mycorrhizal fungi and root-associated microbes, and is driven by carbon from recent photosynthesis and its rhizodeposition (Högberg & Read 2006). A suite of eddy covariance studies across broad ecosystem types demonstrate a tight coupling of GPP and respiration, where interannual changes in carbon sequestration associate with simultaneous increases or decreases in both component fluxes (Baldocchi 2008). Isotopic analyses suggest that heterotrophic respiration also declines for 1–2 years after tree girdling because of the loss of fine root input as respiratory substrate (Bhupinderpal *et al*. 2003). Insect induced mortality cuts off the short term cycling of carbon, such that less carbon is taken up and less is released.

Ultimately, GPP provides all the carbon input for ecosystem metabolism, but substrate for heterotrophic respiration can originate from newly fixed carbon or a variety of stored or decomposing pools of different ages (Ryan & Law 2005). Soil respiration is a composite of heterotrophic and autotrophic driven oxidative fluxes from pools of different ages and chemistry, and the interactions between dominant biotic and abiotic controls vary across different temporal scales (Taneva & Gonzalez-Meler 2011). Infestation driven tree mortality eliminates transpiration and increases soil moisture (Morehouse *et al*. 2008; Clow *et al*. 2011), and the long-term persistence and size of the soil organic matter pool is likely influenced by soil microclimate and edaphic properties interacting with decreased plant production. The patterns we observed at the valley scale suggest that the photosynthetic and respiratory fluxes are tightly though not completely coupled (Fig. 1). The per cent changes in proxy fluxes at the valley scale (Fig. 1) are less pronounced than changes in observed fluxes at the plot scale (Fig. 2) because the valley contains substantial numbers of unaffected Engelmann spruce, subalpine fir, and surviving lodgepole pines and these may benefit from the mortality of competitors. When GPP was removed from plots, we found an initial decline in soil efflux and a partial but transitory recovery after about 5–6 years (Fig. 2a), consistent with an observed increase in K_2_SO_4_ extractable soil DOC (Fig. 2b) and apparently caused by the increased availability of decomposing substrate. This is consistent with regional scale patterns of litter-fall observed post bark-beetle disturbance in Colorado, where the depth of the litter layer was greater 4–7 years after beetle infestation compared to more recently disturbed and undisturbed plots (Klutsch *et al*. 2009). A recent study from Yellow Stone National Park suggests that the timing of the secondary pulse in soil efflux (Fig. 2) linked to older, less labile carbon pools, is likely controlled by the fall rate of litter and the relative impact of post-disturbance water availability and soil temperatures on decomposition processes (Griffin *et al*. 2011). We conclude that the decoupling of GPP and respiration 3–4 years after the peak infestation at the valley scale (Fig. 1) results from the transition from newly fixed carbon to decomposing material as the primary substrate for respiration.

Despite the significance of large scale insect outbreaks to carbon sequestration, the land surface components of the current generation of global climate models cannot recreate episodic biotic disturbances mechanistically (Running 2008) and many models used to predict ecosystem respiration use zero-order kinetics despite clear evidence for substrate limitation (see Ryan & Law 2005; Richardson *et al*. 2006). Current models predict an increase in heterotrophic respiration following insect outbreaks, associated with decaying material (Edburg *et al*. 2011) leading to a large shift from carbon sink to carbon source for these ecosystems (Kurz *et al*. 2008). However, we find no large pulse of respired carbon at the plot or valley scale. The observed loss in soil extractable DOC after the beetle outbreak (Fig. 2b) is the result of reduced input of photosynthetic carbon (Fig. 1a) rather than any increased output of carbon from the respiration of dead material. The secondary decline in plot level respiration (after about 6 years; Fig. 2) and reduction in CO_2_ release at the valley scale (Fig. 1) are difficult to explain without considering decadal effects of substrate limitation on decomposition, described previously only for short periods by others (Bhupinderpal-Shingh *et al*. 2003; Högberg & Read 2006). Our results suggest that any increase in heterotrophic respiration after these types of disturbances is substantially offset by a decline in respiration driven by loss of autotrophic substrate. The overall decrease in total respiration is driven in large part by decreased plant respiration and decreased microbial respiration associated with decreased autotrophic substrate. It has been suggested that models with explicit links between soil organic carbon turnover and microbial processes may be required to effectively model soil carbon release (e.g. Schimel & Weintraub 2003; Zobitz *et al*. 2008; Alison *et al*. 2010). While models that assume respiration rates are proportional to carbon pool size may perform adequately in equilibrium conditions, these models are unlikely to accurately predict carbon cycling after disturbances (Schimel & Weintraub 2003).

Globally we have seen a large increase in the rate and extent of forest mortality in recent years (Allen *et al*. 2010). After insect induced mortality, standing dead trees may remain for several decades (Harmon *et al*. 1986; Edburg *et al*. 2012; Hicke *et al*. 2012). Given the cold temperatures prevalent in mountain forests and the long-term effects of substrate limitation on decomposition, carbon may remain stored in forest ecosystems for long periods after this current, widespread insect outbreak in the absence of rapid release by fire. A study spanning 100 years of fire and beetle disturbance records found no increased occurrence of fire at locations previously infested by beetles (Kulakowski & Jarvis 2011), so release of carbon by decomposition is likely. We have coupled direct experimental manipulations with long-term monitoring to elucidate the mechanisms controlling the release of CO_2_ by decomposition after insect disturbance. In this study, reduced GPP strongly limits respiration after tree mortality and, for up to a decade after the disturbance the current paradigm of simple first order control of ecosystem respiration cannot explain the observed patterns. In addition to studies of how disturbance influences the soil microclimate, it is likely that a more complete treatment of non-equilibrium microbial respiration may be required to confidently model how widespread tree mortality will change long-term carbon balance.
