# Supplementary material for: Persistent reduced ecosystem respiration after insect disturbance in high elevation forests
Source: Ecol Lett. 2013 Mar 17;16(6):731–7. doi: 10.1111/ele.12097 (PMC3674530; doi:10.1111/ele.12097)
Supplement: Supplementary file 1 [file ele0016-0731-SD1.pdf]

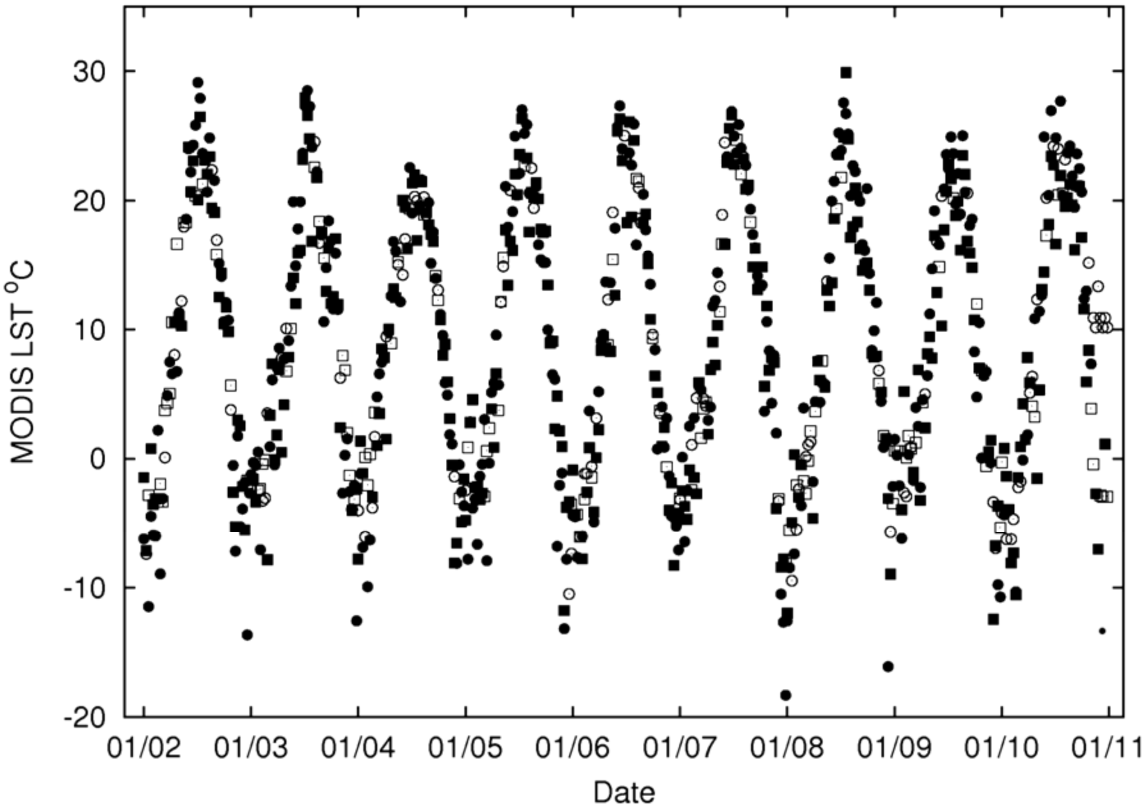

Fig. S1. LST data for MODIS pixels containing the NWT flux tower (squares) and the FEF Head Quarters site (circles). Filled symbols represent actual MODIS observation and open symbols are gap-filled using the temporal window.
