# Supplementary material for: Persistent reduced ecosystem respiration after insect disturbance in high elevation forests
Source: Ecol Lett. 2013 Mar 17;16(6):731–7. doi: 10.1111/ele.12097 (PMC3674530; doi:10.1111/ele.12097)
Supplement: Supplementary file 2 [file ele0016-0731-SD2.pdf]

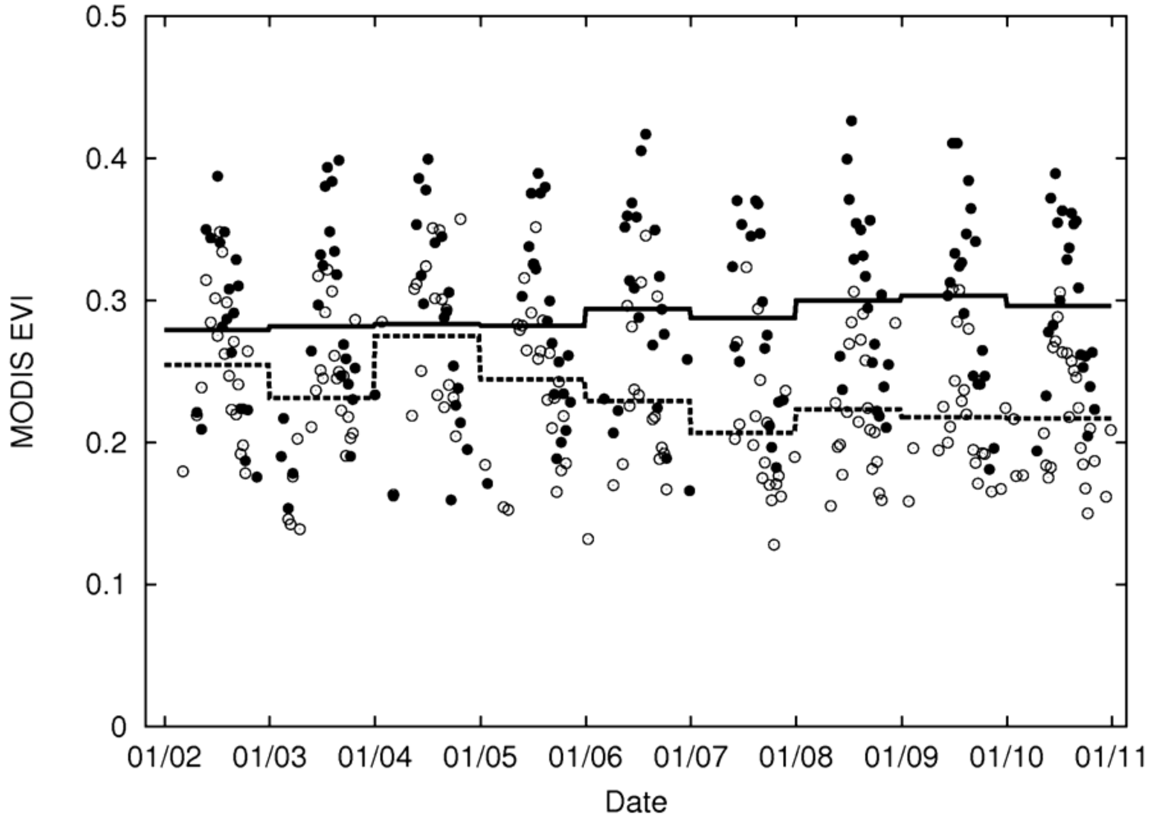

Fig. S2. EVI data for MODIS pixels containing the NWT flux tower (closed circles and solid line) and the FEF Head Quarters site (open circles and dashed line). The symbols represent actual MODIS data and the lines are the annual means.
