# Supplementary material for: Persistent reduced ecosystem respiration after insect disturbance in high elevation forests
Source: Ecol Lett. 2013 Mar 17;16(6):731–7. doi: 10.1111/ele.12097 (PMC3674530; doi:10.1111/ele.12097)
Supplement: Supplementary file 3 [file ele0016-0731-SD3.pdf]

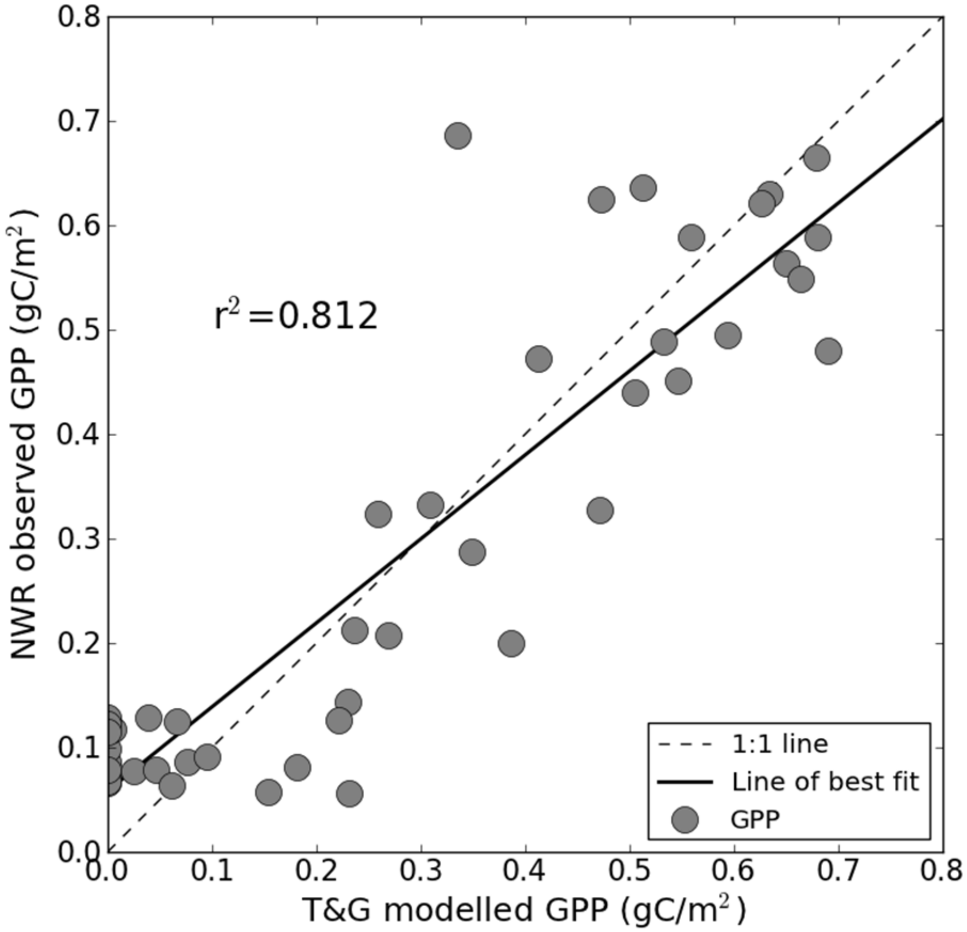

Fig S3: Comparison of GPP observed at NWT and estimated by the T&G model. The data are for 2009 and were not included in the calibration of the model parameters.
