# Supplementary material for: Persistent reduced ecosystem respiration after insect disturbance in high elevation forests
Source: Ecol Lett. 2013 Mar 17;16(6):731–7. doi: 10.1111/ele.12097 (PMC3674530; doi:10.1111/ele.12097)
Supplement: Supplementary file 4 [file ele0016-0731-SD4.pdf]

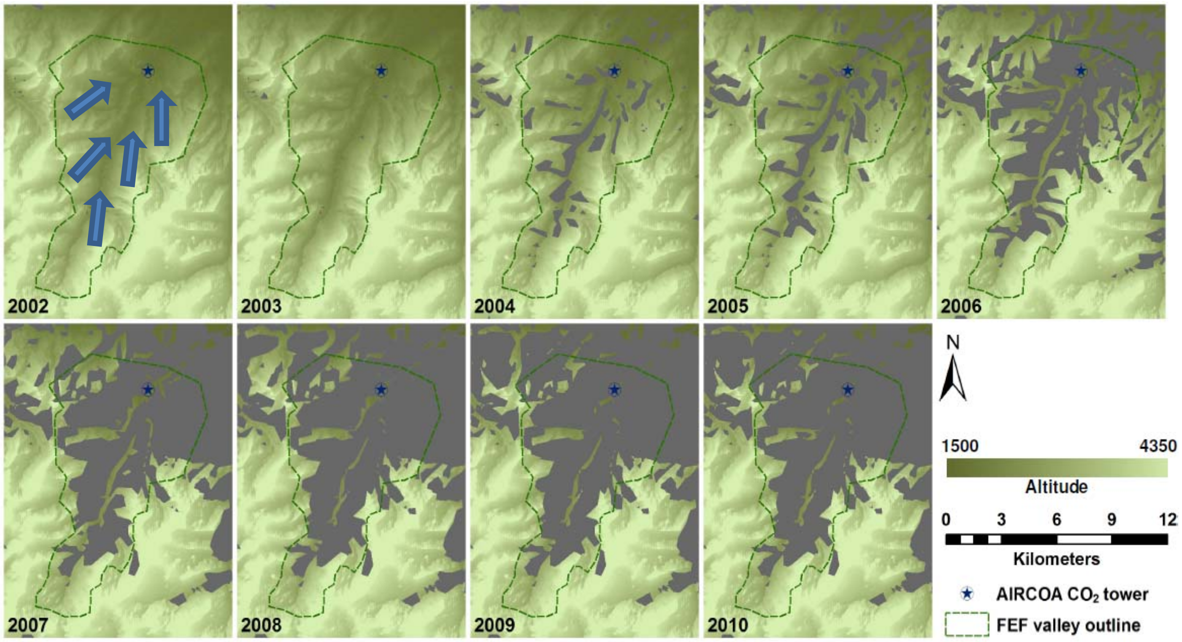

Fig. S4. The study area within the St Louis Creek Valley is outlined with a dotted line, and the AIRCOA CO_2 sensor close to the Fraser Experimental Forest headquarters is indicated with a star. Cumulative estimates of beetle infestation are plotted in dark grey from 2002 through 2010 indicating that the largest annual increase in infestation occurred between 2005 and 2006. Since 2002, the number of live lodgepole pines per hectare, basal area, and quadratic mean diameter have been reduced by 42%, 69%, and 34%, respectively at FEF and surrounding areas (Klutch et al. 2011). Topographic variation is shown by the light grey shading and notional night-time flows are indicated by the blue arrows in the 2002 plot. Infestation estimates are based on the USDA Forest Service, Forest Health Protection and its partners. Forest Insect & Disease Aerial Survey Data Download [Online]. Available from www.fs.usda.gov/goto/r2/fh
