# Supplementary material for: Persistent reduced ecosystem respiration after insect disturbance in high elevation forests
Source: Ecol Lett. 2013 Mar 17;16(6):731–7. doi: 10.1111/ele.12097 (PMC3674530; doi:10.1111/ele.12097)
Supplement: Supplementary file 5 [file ele0016-0731-SD5.pdf]

Table S1: Original and optimized parameter values for the TG model.

	TG original value	Optimized value	Uncertainty (±1σ)
Minimum LST	0°C	-0.9°C	1.3°C
Optimum LST	30°C	22.0°C	1.0°C
Maximum LST	50°C	52.8°C	4.5°C
