# Supplementary material for: Persistent reduced ecosystem respiration after insect disturbance in high elevation forests
Source: Ecol Lett. 2013 Mar 17;16(6):731–7. doi: 10.1111/ele.12097 (PMC3674530; doi:10.1111/ele.12097)
Supplement: Supplementary file 6 [file ele0016-0731-SD6.pdf]

Table S2: Annual sums for GPP_{TG} and mean CO_2 accumulation from Niwot Ridge and Fraser Experimental Forest from 2002 through 2010 (graphed as normalized values in Figure 1a.)

	Gross Primary Productivity		
Year	NWT gC m^{-2} y^{-1}	FEF gC m^{-2} y^{-1}	FEF Valley CO_2 accumulation (Δ ppm d^{-1})
2002	795.4	596.3	.
2003	788.5	507.0	.
2004	859.0	574.3	.
2005	807.1	529.8	.
2006	778.5	542.5	16.36
2007	817.4	471.7	15.42
2008	838.8	449.1	13.29
2009	858.8	441.1	14.33
2010	849.9	522.9	13.94
2011	860.3	452.2	14.94
