# Supplementary material for: Persistent reduced ecosystem respiration after insect disturbance in high elevation forests
Source: Ecol Lett. 2013 Mar 17;16(6):731–7. doi: 10.1111/ele.12097 (PMC3674530; doi:10.1111/ele.12097)
Supplement: Supplementary file 7 [file ele0016-0731-SD7.pdf]

Table S3: Mean growing season soil efflux (µmol CO_2 m^{-2} s^{-1}) and soil total organic carbon (µgC g-1 dry soil) from plots across the NWT gridling and FEF beetle-kill chronosequences. Standard error of the mean values are show in brackets, n=3 for plots with killed trees and n=6 for plots with live trees. (graphed as normalized values in Figure 2)

Site	Year of Mortality	Soil efflux (µmol CO_2 m^{-2} s^{-1})	Soil Total Organic Carbon (µgC g-1 dry soil)
NWT	Live trees	3.62 (0.22)	775.8 (109)
FEF	Live trees	3.29 (0.18)	1004 (77)
NWT	2010	2.9 (0.30)	735.3 (109)
NWT	2009	2.43 (0.30)	547 (109)
FEF	2008-09	1.93 (0.23)	253.5 (133)
NWT	2008	2.52 (0.30)	530.4 (109)
FEF	2004-05	3.17 (0.21)	633.1 (133)
NWT	2004	3.24 (0.30)	785.4 (109)
NWT	2003	2.6 (0.30)	674.9 (109)
FEF	2002-03	2.47 (0.22)	438.7 (133)
NWT	2002	2.74 (0.30)	584.3 (109)
