# Supplementary material for: Persistent reduced ecosystem respiration after insect disturbance in high elevation forests
Source: Ecol Lett. 2013 Mar 17;16(6):731–7. doi: 10.1111/ele.12097 (PMC3674530; doi:10.1111/ele.12097)
Supplement: Supplementary file 8 [file ele0016-0731-SD8.pdf]

Appendix S1: *Supplemental analysis to illustrate the method to estimate relative changes in valley respiration from night time accumulation of CO_2 at FEF*

Title: **Persistent reduced ecosystem respiration after insect disturbance in high elevation forests**

Contrary to expectations we find systematic decreases in ecosystem respiration after mountain pine beetle outbreaks in western U.S. forests using valley CO_2 monitoring and chronosequences of natural and induced tree mortality spanning a nine-year period following disturbance.

Short title: Reduced Respiration after Insect Outbreaks

Classification: BIOLOGICAL SCIENCES: Ecology; Environmental Sciences

Authors: David J.P. Moore[a*], Nicole A. Trahan[b*], Phil Wilkes[c], Tristan Quaife[d], Britton B. Stephens[e], Kelly Elder[f], Ankur R. Desai[g], Jose Negron[f], Russell K. Monson,[a,h]

[a]School of Natural Resources and the Environment, University of Arizona, Tucson, AZ
[b]Department of Ecology and Evolutionary Biology, University of Colorado at Boulder, Boulder, CO USA
[c]School of Mathematical and Geospatial Sciences, RMIT, GPO Box 2476, Melbourne, Victoria, 3001, Australia
[d]Department of Meteorology, University of Reading, Reading, RG6 6BB, United Kingdom.
[e]National Center for Atmospheric Research, Boulder, CO, USA
[f]US Forest Service, Rocky Mountain Research Station, 240 W Prospect Road Fort Collins, CO USA
[g]Dept of Atmospheric & Oceanic Sciences, University of Wisconsin, Madison, WI USA
[h] Laboratory for Tree Ring Research, University of Arizona, Tucson, AZ USA

[*] These authors contributed equally to this work
Correspondence to: David JP Moore, (current address) School of Natural Resources and Environment. 1955 E. 6th Street, University of Arizona, Tucson, AZ 85721

E-mail:davidjpmoore@email.arizona.edu

Keywords: Gross primary productivity, ecosystem respiration, Mountain pine beetle, subalpine forest, carbon balance, insect outbreak, Mountain West, lodgepole pine, disturbance

Supplemental analysis to illustrate the method to estimate relative changes in valley respiration from night time accumulation of CO_2 at FEF

Night-to-night variations depend primarily on wind-speed and direction (Fig. S5), but seasonal variations in the extent of CO_2 build-up (Fig S6) are driven primarily by soil-temperature effects on respiration. However, once corrected for the time dependent increase in global and regional CO_2 concentration (Fig S7), we find the seasonal peak in soil temperature has remained constant while the seasonal peak in CO_2 build-up has decreased, suggesting that soil temperature is not driving the observed changes in peak build-up from year to year (Fig S8). This interpretation assumes that inter-annual variations in night-time wind speed and mixing within the valley are not significant. This assumption is justified through an analysis of proximate measures of the boundary layer conditions within the valley (Fig S9, S10). If there were a systematic change in the nocturnal drainage flow, we would expect to see trends in the vertical stability gradient within the valley, as the strength and depth of a stable layer is related to this quantity (Stull 1988) or in the wind speeds themselves. However, we find no significant difference of variation in stability with time over Jun-Aug 2007-2010 (when temperature gradient observations were available) (Figure S9). Also there is no change in median wind speed at the Fool Creek station and only a very small (0.06 m/s) change in median wind speed at the Headquarters station (Fig S10), furthermore the year-to-year variations in wind speed do not correlate with variations in CO_2 build-up which supports a biological driver for the changes in CO_2 build-up (compare Fig S10 and S9b). The annual sums of night time CO_2 accumulation are shown in Table S2.

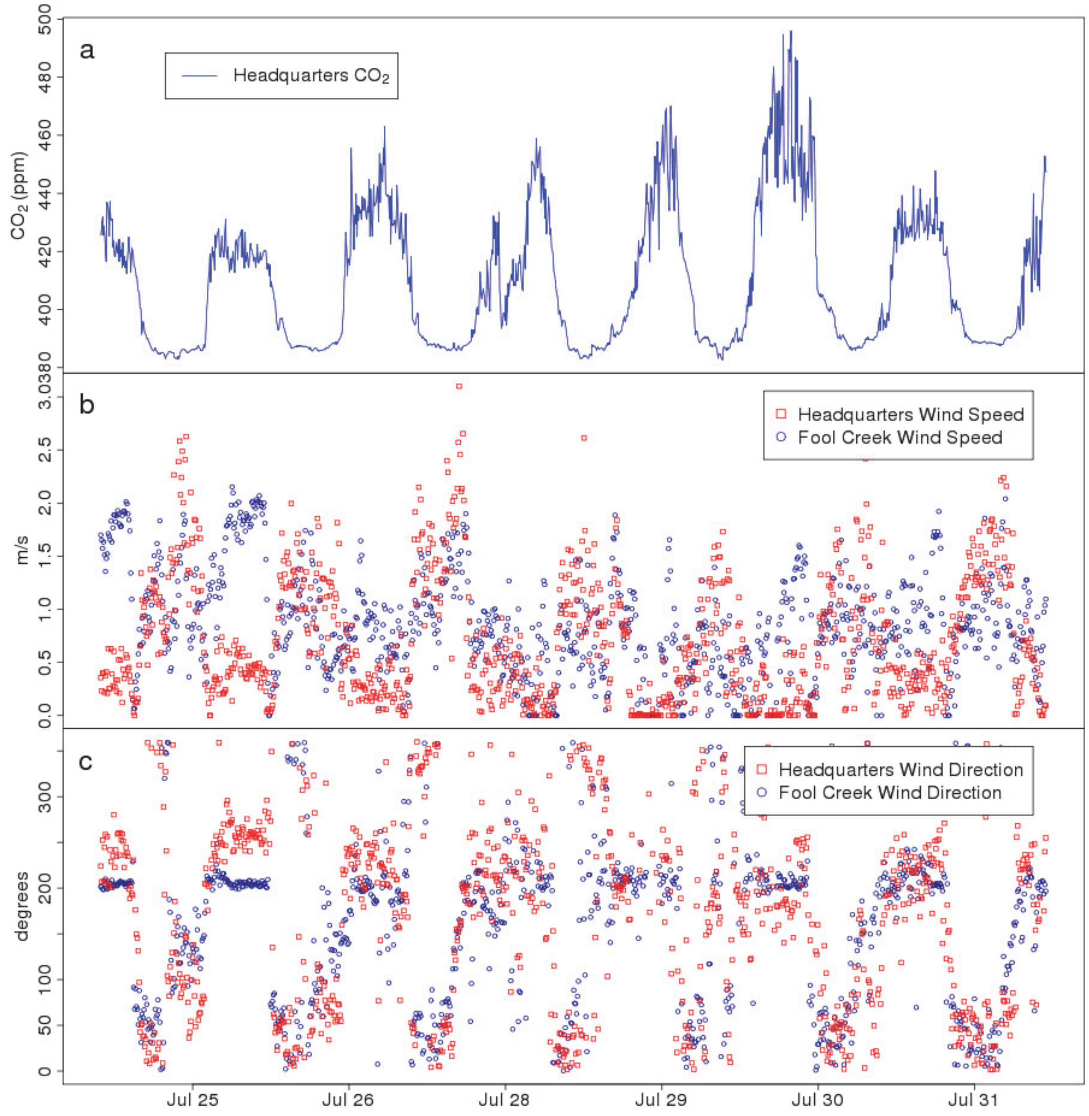

Figure S5. Hourly-average (a) CO_2 concentrations from the FEF Headquarters tower for one week in July, 2009 along with (b) wind-speed and (c) wind direction for Headquarters and for a tower at 3100 m on the Fool Creek tributary to St. Louis Creek. Dates are shown at midnight local time.

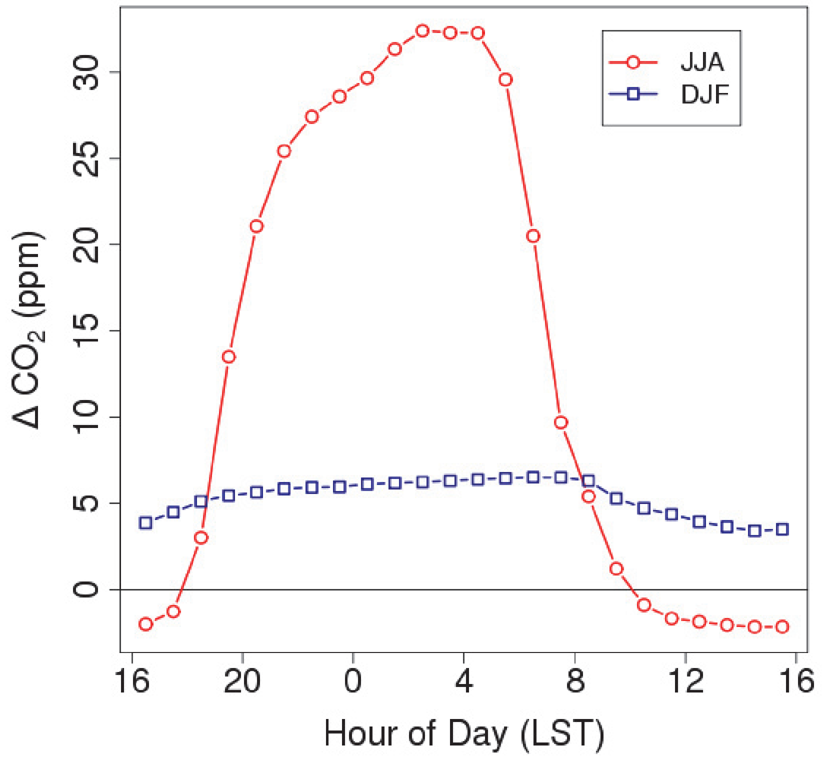

Figure S6. Average diurnal CO_2 concentrations measured at FEF during June, July, and August or December, January, and February. A common deseasonalized, smoothed, reference time series from Mauna Loa Observatory, Hawaii (NOAA ESRL) has been subtracted from the data to allow the combination of data from multiple years.

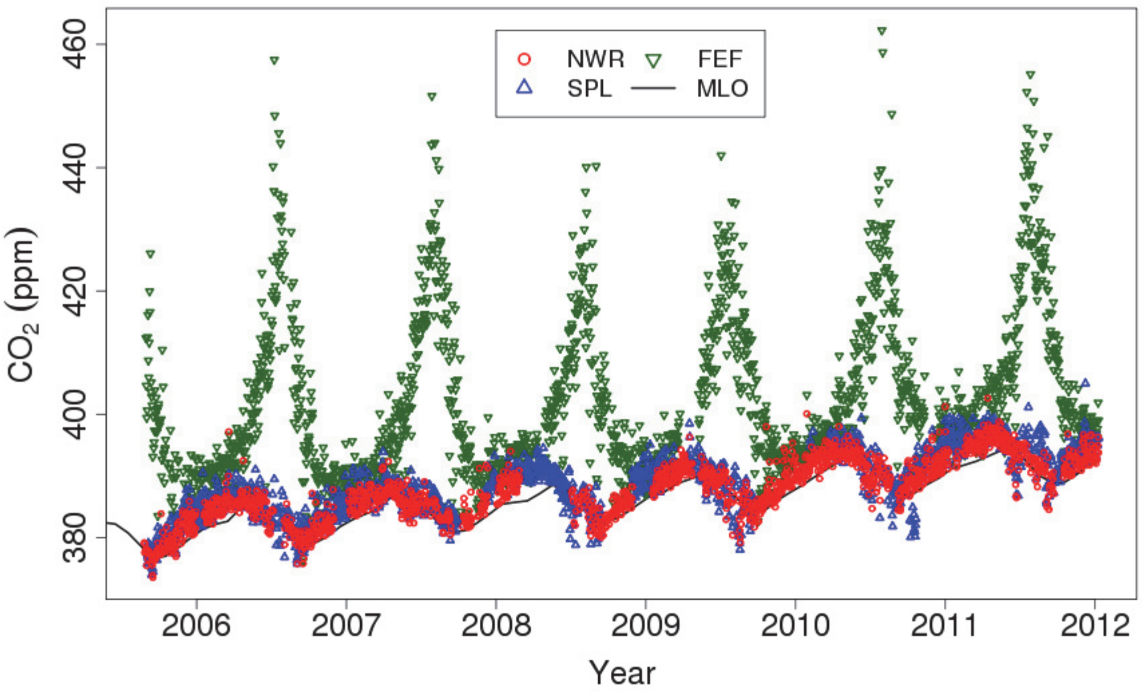

Figure S7. Nightly (0000-0400 LST) average CO_2 concentrations at FEF and in stable-CO_2 conditions at NWT, and a third mountain-top site at Storm Peak Laboratories Laboratory (SPL) in Steamboat Springs, CO. Monthly mean data from Mauna Loa Observatory (MLO, NOAA ESRL) in Hawaii are also shown for reference.

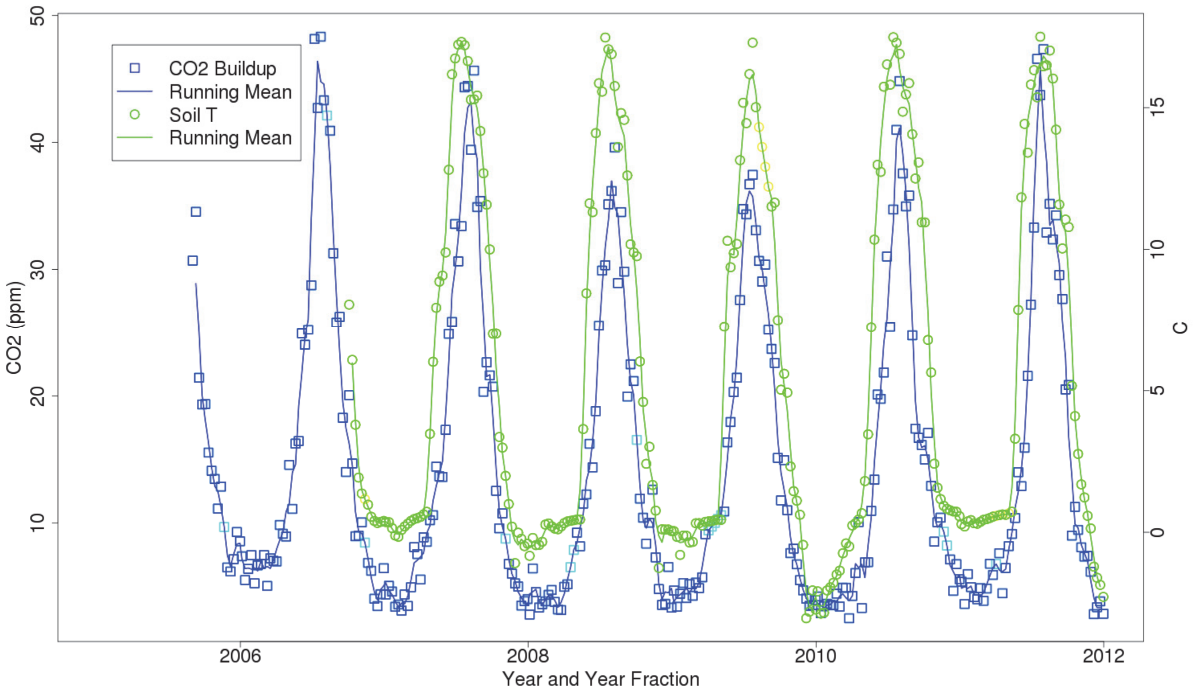

Figure S8. 8-day average nightly CO_2 buildup as shown in Figure 1b along with corresponding average soil temperature from 5 cm at the Headquarters site over the period of soil temperature data availability. CO_2 build-up symbols in cyan and soil temperature symbols in yellow have been interpolated.

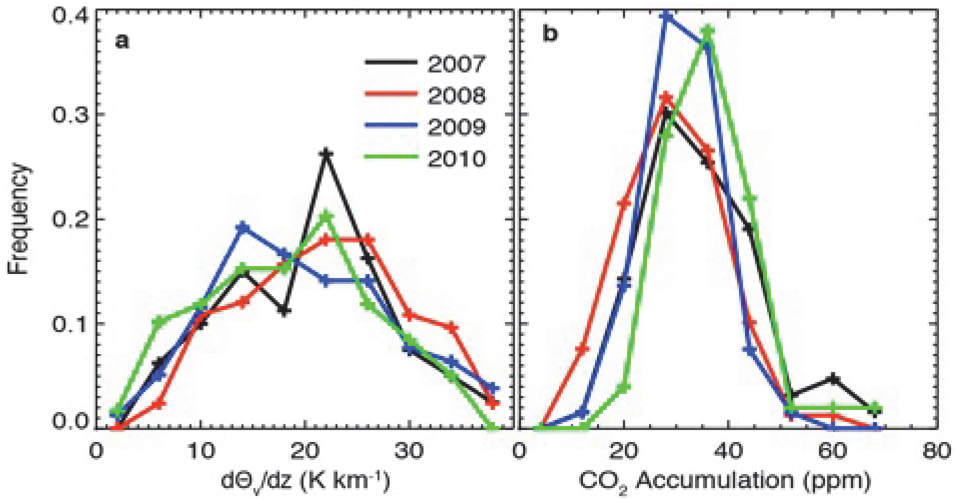

Figure S9. a) Seasonal histograms of night average virtual potential temperature gradient between a tower near valley base (Headquarters, 2760 m) and along the valley wall (Fool Creek, 3100 m) for years when data at both towers were available reveals a stationary frequency distribution of this gradient indicating no significant change in nocturnal boundary layer strength, which is in contrast to b) showing significant changes in the histogram of nocturnal background-corrected CO_2 build-up observed by the AIRCOA sensor.

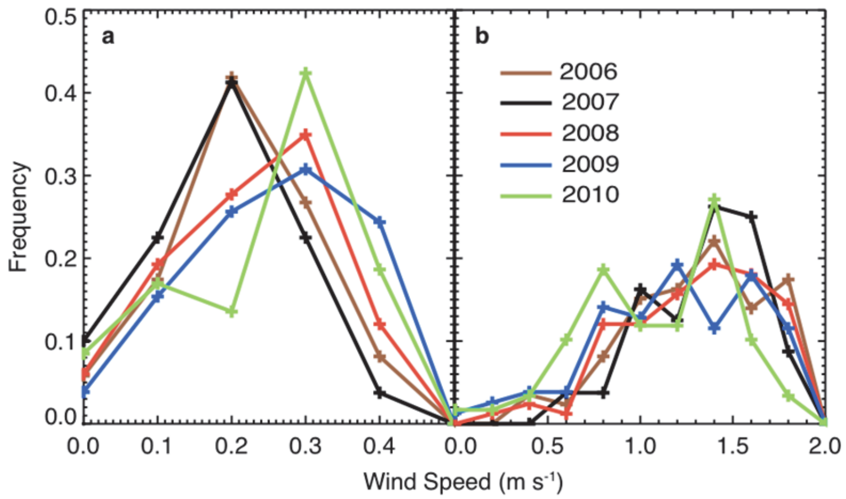

Figure S10. Frequency histograms of night-time windspeed during periods of down valley flow for the Headquarters (a) and Fool Creek meteorological stations from 2006 through 2010.

References (not in main text):

Stull, R.B. *An Introduction to Boundary-Layer Meteorology*. Dordrecht, Netherlands: (Kluwer Academic Publishers, 1988), pp 670.
